# Spermidine restores dysregulated autophagy and polyamine synthesis in aged and osteoarthritic chondrocytes via EP300

**DOI:** 10.1038/s12276-018-0149-3

**Published:** 2018-09-19

**Authors:** Pradeep K. Sacitharan, Seint Lwin, George Bou Gharios, James R. Edwards

**Affiliations:** 1 Botnar Research Centre, University of Oxford, Oxford, UK; 2 The Institute of Ageing and Chronic Disease, University of Liverpool, Liverpool, UK

## Abstract

Ageing is the primary risk factor for osteoarthritis (OA). A decline in the ageing-associated process of autophagy is suggested as a potential contributor to OA development. Polyamines such as spermidine decrease during ageing, contributing to impaired autophagy and reduced cellular function. However, the role of polyamines and their effect on the regulatory mechanism governing autophagy in aged and arthritic cartilage tissue has not been established. Elucidating if polyamine regulation of autophagy is impaired during ageing and OA in chondrocytes may lead to improved treatment approaches to protect against cartilage degradation. Our results indicate that polyamine synthesis was decreased in aged and OA cartilage, along with reduced autophagy activity, evidenced by decreased autophagy-related gene and protein expression and autophagosome formation. Importantly, spermidine treatment increased the expression of the acetyltransferase EP300, which binds to crucial autophagy proteins, Beclin1 and LC3, and elevates chondrocyte autophagy. Our data indicate spermidine prevents the ageing- and OA-related decrease in autophagy and may protect against OA development.

## Aging: toward a natural treatment for osteoarthritis

The naturally occurring small molecule spermidine might prevent and repair the cartilage damage in osteoarthritis, commonly associated with aging. Spermidine was first found in semen but is widely distributed in animals and plants and is known to promote longevity in mammals. During normal aging, a process of “autophagy” in which cells degrade and recycle used materials is known to decline in chrondrocytes, cells which produce and maintain cartilage. Researchers in the UK led by James Edwards at the University of Oxford have uncovered details of the biochemical mechanisms linking spermidine and autophagy. Treating cultured human and mouse chrondrocytes with spermidine can reverse the decline in autophagy. The researchers identified specific genes and proteins involved in spermidine’s effects. Spermidine treatment might restore normal autophagy, combatting osteoarthritis.

## Introduction

Osteoarthritis (OA) is the most common form of arthritis characterised by cartilage degradation, synovitis and pain^1^. There are no effective therapeutic options for OA^1^. The primary risk factor for OA is increased age, where chondrocytes, the only resident cells in cartilage, fail to maintain extracellular matrix turnover and cartilage integrity diminishes over time^2^. The well-conserved cellular process of autophagy (self-eating), where unwanted or dysfunctional cytoplasmic constituents are isolated, degraded and recycled, decreases with age, leading to an accumulation of toxic and non-functional protein elements^3^. Autophagy decreases in OA^4,5^ and mice deficient in the key autophagy protein ATG5, have been shown to develop premature OA^6^. Spermidine an organic polyamine, found in peas and whole grains, has previously been shown to enhance autophagy and protect against age-related pathologies^7^. However, the mechanistic regulation of autophagy by spermidine is not fully understood and the role of polyamines in OA is not established. Previously, the regulation of the epigenetic modifier and acetyltransferase EP300, has been linked to changes in autophagic flux, and where inhibition of EP300 by spermidine treatment may influence normal cell biology through post-translational modifications of essential autophagy-related protein complexes^8,9^. Importantly, the role of EP300 in chondrocytes is also not established. This study investigates the expression and autophagy-related protein interactions in ageing- and OA-cartilage and whether spermidine-induced EP300 regulation might provide a mechanism through which chondrocyte autophagy in aged cartilage and OA might be reactivated.

## Material and methods

### Isolation of human chondrocytes

Healthy or OA knee/hip cartilage was obtained from surgical patients. Tissue samples were collected with informed donor consent in full compliance with national and institutional ethical requirements, the United Kingdom Human Tissue Act, and the Declaration of Helsinki. Dissected cartilage pieces were incubated overnight in DMEM with 1 mg/ml Collagenase A (Roche Pharmaceuticals) at 37 °C for 5–6 h to isolate cells. Cells used in experiments were at passage 1.

### Murine studies

Wild-type young (2 months) or old (16 months) C57BL/6 mice (Jackson Laboratory) were used. All animal procedures were approved by the British Home Office, under the United Kingdom Animal (Scientific Procedures) Act 1986. Femoral heads of mice were avulsed and placed in culture media, Dulbecco’s modified Eagle’s medium (DMEM), for ex vivo experiments.

### Cell culture

Isolated human chondrocytes and HTB-94 chondrosarcoma cells (American Type Culture Collection) were cultured in DMEM containing 4.5 g/l of glucose and L-glutamine (Lonza), 10% foetal calf serum (PAA Laboratories), 1% Penicillin and Streptomycin (Cambrex), Amphotericin B (Gibco) and HEPES solution (Cambrex) unless otherwise stated.

### siRNA transfection

Healthy isolated human chondrocytes were wet reverse transfected with Dharmacon ON-TARGETplus SMARTpool (4 oligos) human EP300 siRNA (25 nM) or ON-TARGETplus EP300 siRNA (Dharmacon Technologies) for 24 h. A scramble oligo sequence was used as control (25 nM) (Dharmacon Technologies).

### Drug treatments

Cells were seeded at 300,000 cells per well in six-well plates in serum starved media for 1 h then washed. Thereafter, cells were seeded in complete DMEM media treated with either spermidine (100 nM; Sigma-Aldrich) or the control of DMSO (Sigma-Aldrich) for 2 h. For fluorescence-activated cell sorting (FACS) experiments, Bafliomycin A1 (BAF; 10 nM; Sigma-Aldrich) was used alongside other treatments as a positive control. For green fluorescent protein (GFP) studies, groups of cells were seeded in serum starved media alongside drug treatments for positive controls. To evaluate the effect of spermidine on EP300, human chondrocytes were transfected with EP300 siRNA for 24 h then treated with spermidine (100 nM) for 2 h.

### Fluorescence-activated cell sorting

Autophagy was also measured by quantifying Microtubule-associated proteins 1 A/1B light chain 3B (LC3)-II mean fluorescence intensity using the FlowCellect Autophagy LC3 Antibody-based Assay Kit (Merck-Millipore) according to the manufacturer’s instructions after treating the cells with either spermidine (100 nM), the vehicle control of DMSO or/and BAF (10 nM). Untreated cells were also used as a negative control. Use of this kit includes a step where cytosolic LC3-I is washed from the cell, leaving only membrane bound LC3-II prior to staining.

### LC3-GFP studies

LC3-GFP murine tissues were generously gifted by Prof Katja Simon (University of Oxford). These commercially available transgenic mice were previously described by Mizushima^10^. Hips from LC3-GFP mice were avulsed and placed in DMEM media containing either spermidine (100 nM) or DMSO. For positive controls, serum free DMEM containing either spermidine (100 nM) or DMSO was used. After an incubation of 2 h, hips were embedded in Optimal Cutting Temperature (CellPath) and snap-frozen. Thereafter, samples were cryosectioned at 10 µm. Fluorescence images were taken using an excitation wavelength of 473 nm and a band-path of 490−540 nm. Bright field images were obtained using scattering wavelength of 635 nm. The cells were segmented in bright field, and GFP intensity was measured in fluorescent channel. Intensity of background was subtracted from intensities of individual cells and divided by intensity of background in order to normalise and score cells. The number of LC3 puncta per cell was counted throughout each z stacks by two blinded operators.

### Protein analysis

Cells were homogenized in lysis buffer (RIPA buffer) (Sigma-Aldrich), Ethylenediaminetetraacetic acid free protease inhibitor (Roche Pharmaceuticals), Phosphatase inhibitor cocktail 2 and 3 (Sigma-Aldrich) and protein levels quantified by bicinchoninic acid assay (Thermo Fisher Scientific). Lysed protein samples were probed overnight for western blotting with primary antibody: E1A-associated protein p300 (EP300) (Novus Biologicals), Beta (β)-actin (Sigma-Aldrich), Serine/threonine-protein kinase (ULK1) (Novus Biologicals), Beclin1 (Novus Biologicals), LC3 (Novus Biologicals), Acetylated-Lysine (Cell Signalling Technology), Type II collagen (COL2A1) (Sigma-Aldrich) or Sex determining region Y-box9 (SOX9) (Abcam); Nucleoporin p62 (p62) (Novus Biologicals). Murine hips were avulsed and placed in ice cold protein lysis buffer (prepared as above). Samples were shaken for 2 h at 4 °C and the supernatants collected for western blot analysis as outlined above.

### RNA isolation and quantitative PCR

Total ribonucleic acid (RNA) was isolated from cell cultures using RNeasy Mini Kit (Qiagen) as per manufacturer’s instructions. Microdissected cartilage pooled from six mouse knee joints was collected, placed in TRIzol® (Life technologies) and homogenised using PowerGen™ Model 125 Homogenizer (Fisher Scientific). Thereafter the RNeasy Mini Kit protocol was followed. Thereafter, a High Capacity reverse transcription complementary deoxyribonucleic acid (cDNA) kit (Applied Biosystems) according to the recommendations of the manufacturer was used. Real-time-quantitative polymerase chain reaction (RT-qPCR) was carried out on a ViiA™ 7 System (Applied Biosystems). Relative gene expression of messenger RNA (mRNA) was analysed by the ^∆∆^Ct method using 18s as an endogenous control gene (Table 1). A total of 100 μl reaction mixture containing 50 μl cDNA template (500 ng starting RNA) was used as previously described^11^.

**Table 1:** Primers used in RT-qPCR experiments

Gene	Human	Murine
*18S*	Hs03003631_g1	Mm02601776_g1
*ACAN*	Hs00153936_m1	Mm00545794_m1
*ATG5*	Hs00169468_m1	Mm00504340_m1
*ATG7*	Hs00893766_m1	Mm00512209_m1
*BECN1*	Hs00177504_m1	Mm01265461_m1
*COL2A1*	Hs00264051_m1	Mm01309565_m1
*LC3*	Hs01076567_g1	Mm00458724_m1
*SSAT*	Hs00971739_g1	Mm00485911_g1
*SPDS*	Hs00171253_m1	Mm 00445061_m1
*ODC1*	Hs00159739_m1	Mm02019269_g1
*POA*	Hs00382210_m1	Mm00464096_m1
*EP300*	Hs00914223_m1	Mm00625535_m1
*SOX-9*	Hs01001343_g1	Mm00448840_m1
*ULK1*	Hs00177504_m1	Mm00437238_m1

*ACAN* aggrecan, *ATG5* autophagy related 5, *ATG7* autophagy related 7, *BECN1* Beclin1, *SSAT* spermidine/spermine N-1 acetyl transferase, *SPDS* spermidine synthase, *ODC1* ornithine decarboxylase 1, *POA* polyamine oxidase

### Electron microscopy

Microdissected cartilage from mice was fixed using 2.5% glutaraldehyde + 4% paraformaldehyde (stocks solutions both from Agar Scientific) in 0.1 M 1,4 Piperazine bis (2-ethanosulfonic acid) (Sigma-Aldrich) buffer at pH 7.2 at room temperature for 1–2 h, then at 4 °C until further processing. Samples were prepared as described previously [10] and imaged on an FEI company Tecnai 12 TEM with a Gatan US1000 camera. Thereafter, autophagosomes from images were counted from cells.

### Immunohistochemistry

Heat mediated antigen retrieval was performed on paraffin embedded sections using citric acid buffer (Sigma-Aldrich) warmed in a water bath at 100 °C for 20 mins. LC3 (Novus Biologics) antibody and rabbit Immunoglobulin G (IgG) control (Santa Cruz Biotechnology) were used at 1:200 dilution. Sections were counter stained with Mayers Haematoxylin (Sigma-Aldrich). Slides were imaged using a light microscope (Olympus) at the magnification of x20. Thereafter, quantification of LC3 positive cells/total cells were counted.

### Immunoprecipitation

EP300 or autophagy-related proteins were immunoprecipitated after 24 h treatment of HTB-94 cells treated spermidine (100 nM) or DMSO (control) using a commercially available immunoprecipitation kit (Pierce). Total acetyl lysine or autophagy targets for the respective experiments were detected by western blotting. β-Actin from input whole lysate was used as loading control.

### Statistical analysis

All data are expressed as mean ± standard error of mean (S.E.M) *of n* observations. Experiments were statistically analysed utilising the Students unpaired *t*-test for parametric data with independent groups compared with their specific controls or time-matched controls. One-way analysis of variance (ANOVA) with the Bonferroni post hoc test or ANOVA with Tukeys comparison test was used to compare three or more groups. A significant difference was accepted when *p* < 0.05, *p* < 0.01, *p* < 0.001 or *p* < 0.0001 represented in all tables and Figures as *, **, *** or **** respectively. *NS*, non–significant. Data analysis was performed using GraphPad Prism® 5.0 (GraphPad Software, California, U.S.A).

## Results

### Decreased autophagy and polyamine synthesis in aged and OA human and murine cartilage

**Fig. 1:**
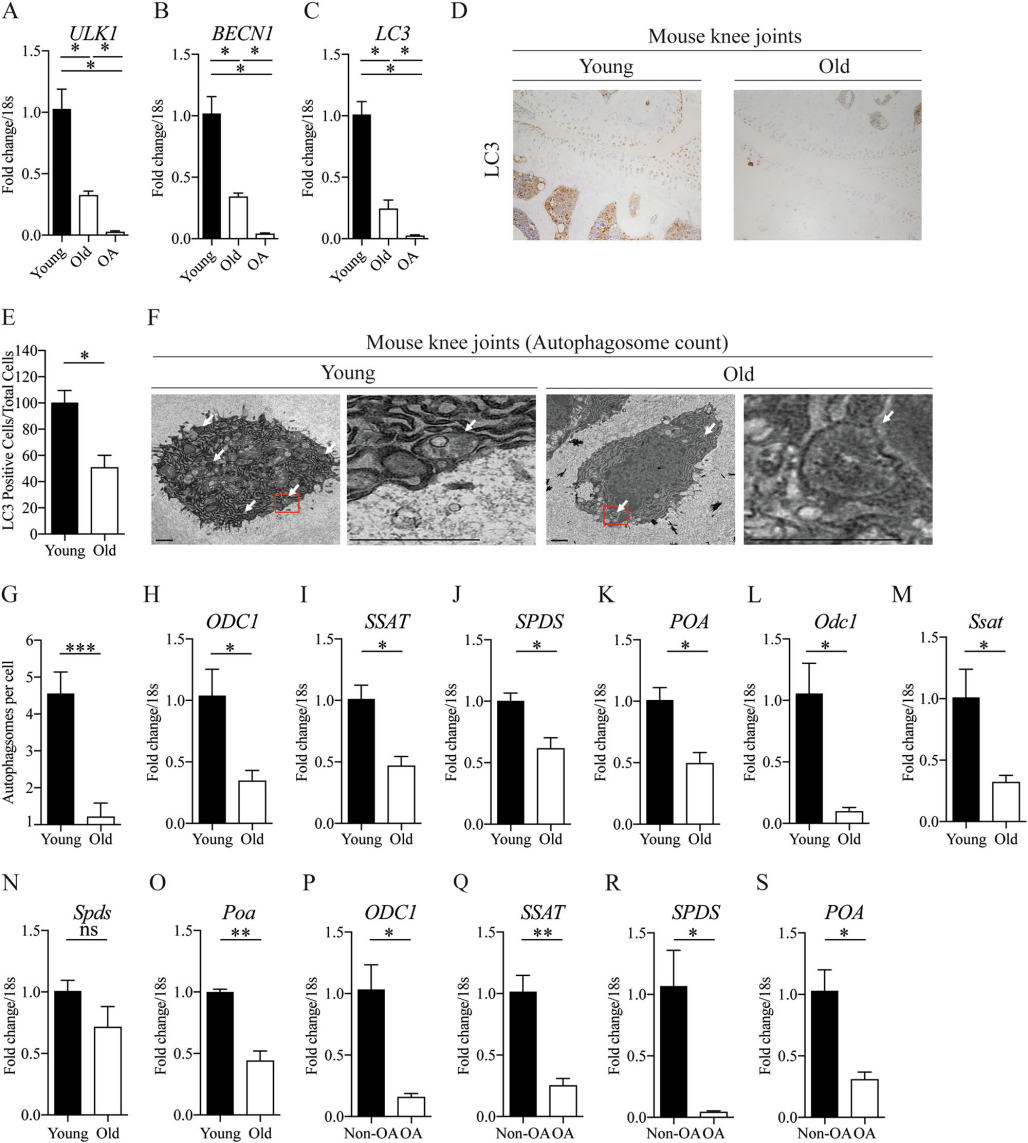
Decreased autophagy and polyamine synthesis in aged and OA human and murine cartilage. **a**–**c** Autophagy gene expression in isolated chondrocytes from young (21–37 years), old (62–68 ) and OA (49–86 years) knee joints (*n* = 3). **d** Representative images (x20) of LC3 protein expression in knee joints of WT mice at 2 months (young) or 16 months (old) of age. **e** Quantification of LC3 positive cells/total from murine knee joints (*n* = 4). **f** Representative EM images of chondrocytes from microdissected cartilage obtained from knee joints of WT young (2 months) or old (16 months) mice. White arrows highlight autophagosomes. Red boxes highlight the area of magnification. Scale bars: 1 μm. **g** Quantification of autophagosomes in chondrocytes (microdissected cartilage) from murine knee joints (*n* = 18 cells per mouse. six mice used). **h**–**k** RT-qPCR analysis gene expression of key enzymes involved in polyamine synthesis from isolated chondrocytes from young healthy (21–37 years) or old (62–68 years) (*n* = 3). **l**–**o** RT-qPCR analysis gene expression of key enzymes involved in spermidine synthesis in microdissected cartilage obtained from knee joints of WT young (2 months) or old mice (16 months) (*n* = 3). **p**–**s** RT-qPCR analysis of gene expression of key enzymes involved in spermidine synthesis in non-OA and OA human chondrocytes (*n* = 3). All RT-qPCR gene expressions were normalised to the endogenous level of 18s. All data are expressed as mean ± S.E.M of *n* observations. Students unpaired *t*-test was used for statistical analysis. *p* < 0.05, *p* < 0.01 or *p* < 0.001 represented in all tables and Figures as *, **, or ***, respectively. NS non–significant

Normal human cartilage samples from aged individuals showed decreased expression of autophagy-related genes Ulk1, beclin1 and LC3 compared with samples of young cartilage. Autophagy-related protein expression was further reduced in OA samples compared to normal controls (Fig. 1a–c and Supplementary Figure 1a). Immunohistochemistry confirmed a similar decrease in LC3 protein expression within articular chondrocytes along with decreased autophagosome formation by scanning electron microscope in cartilage from aged mice compared to young control animals (Fig. 1d–g and Supplementary Figure 1b). Importantly, the mRNA expression of key rate limiting enzymes responsible for polyamine synthesis was also reduced, correlating with the decrease in autophagy levels seen in the same samples (Fig. 1h–s).

### Spermidine activates chondrocyte autophagy

**Fig. 2:**
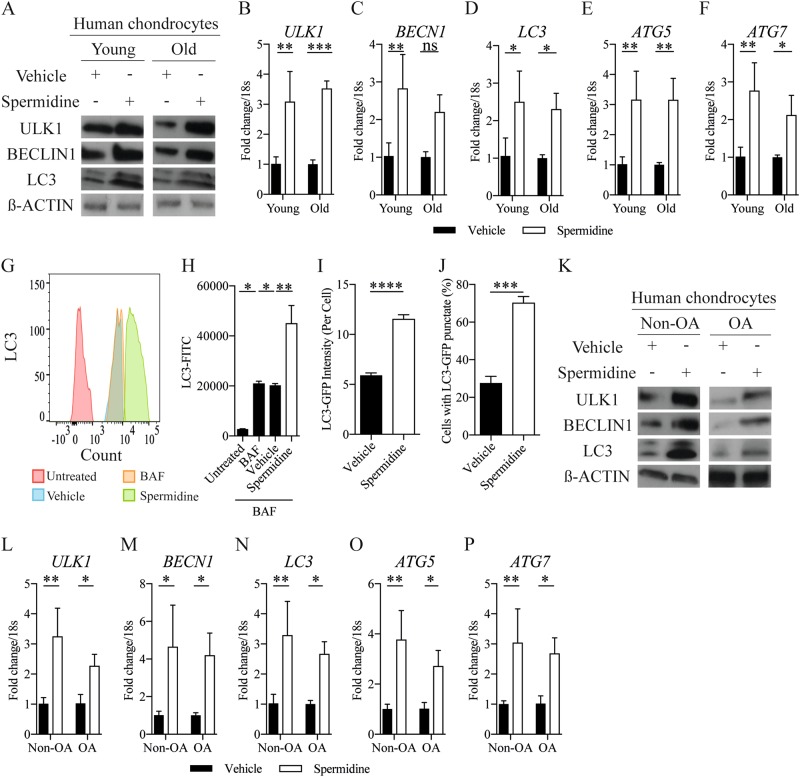
Spermidine activates chondrocyte autophagy. (**a**) Autophagy protein expression and (**b**–**f**) autophagy gene expression (RT-qPCR) in isolated chondrocytes from young (21–37 years) and old (62–68 years) human knee joints treated with DMSO control or spermidine (100 nM) (*n* = 3). **g** Histogram and (**h**) quantification of MFI of LC3-II in HTB-94 cells either untreated or treated with BAF (10 nM), alongside DMSO (vehicle control) or spermidine (100 nM), for 2 h (*n* = 3). **i** Total LC3-GFP intensity and (**j**) quantification of percentage of chondrocytes with LC3 positive punctate per chondrocyte obtained from avulsed femoral heads of LC3-GFP mice. Femoral heads were treated with either DMSO control or spermidine (100 nM) for 2 h before fixation (*n* = 15–37 cells from three mice per treatment group). **k** Protein expression and (**l**–**p**) gene expression (RT-qPCR) of key autophagy proteins in isolated chondrocytes from non-OA control or OA human knee joints. Cells were either treated with DMSO control or spermidine (100 nM) for 2 h (*n* = 3). All RT-qPCR gene expressions were normalised to the endogenous level of 18s. All data are expressed as mean ± S.E.M of *n* observations. Students unpaired *t*-test or ANOVA with Tukeys comparison were used for statistical analysis. *p* < 0.05, *p* < 0.01, *p* < 0.001 or *p* < 0.0001 represented in all tables and Figures as *, **, *** or ****, respectively. NS  non–significant

To determine whether stimulation with spermidine might activate autophagy in this system, protein and gene expression of critical autophagy-related molecules (ULK1, BECLIN1, LC3, ATG5 and ATG7) were examined following spermidine treatment. In both human and murine isolated chondrocytes, autophagy markers were significantly elevated compared to vehicle-treated control cells, this occurred in chondrocytes from both aged and young cartilage (Fig. 2a–f and Supplementary Figure 2a–f). Whereas, P62 (indicates impaired autophagy) protein expression decreased in young and old human isolated chondrocytes after spermidine stimulation (supplementary Figure 2g). The conversion from LC3-I to LC3-II is indicative of increased autophagy^12^. LC3 II mean fluorescent intensity increased in chondrocytes treated with spermidine (Fig. 2g and h). This finding was supported using LC3-GFP reporter mice, where isolated primary chondrocytes showed increased LC3-GFP + puncta (95.37%) and staining intensity per cell (154.17%) following spermidine stimulation (Fig. 2i–j and Supplementary Figure 2h-i). We also observed spermidine also increased protein and gene expression of key autophagy molecules in human isolated chondrocytes from OA patients compared to untreated control cells (Fig. 2k–p). Collectively, this suggests that the decline in chondrocyte autophagy and cellular function with ageing and OA might be reversible depending on the correct stimuli.

### Spermidine increases the expression of key chondrogenesis markers

**Fig. 3:**
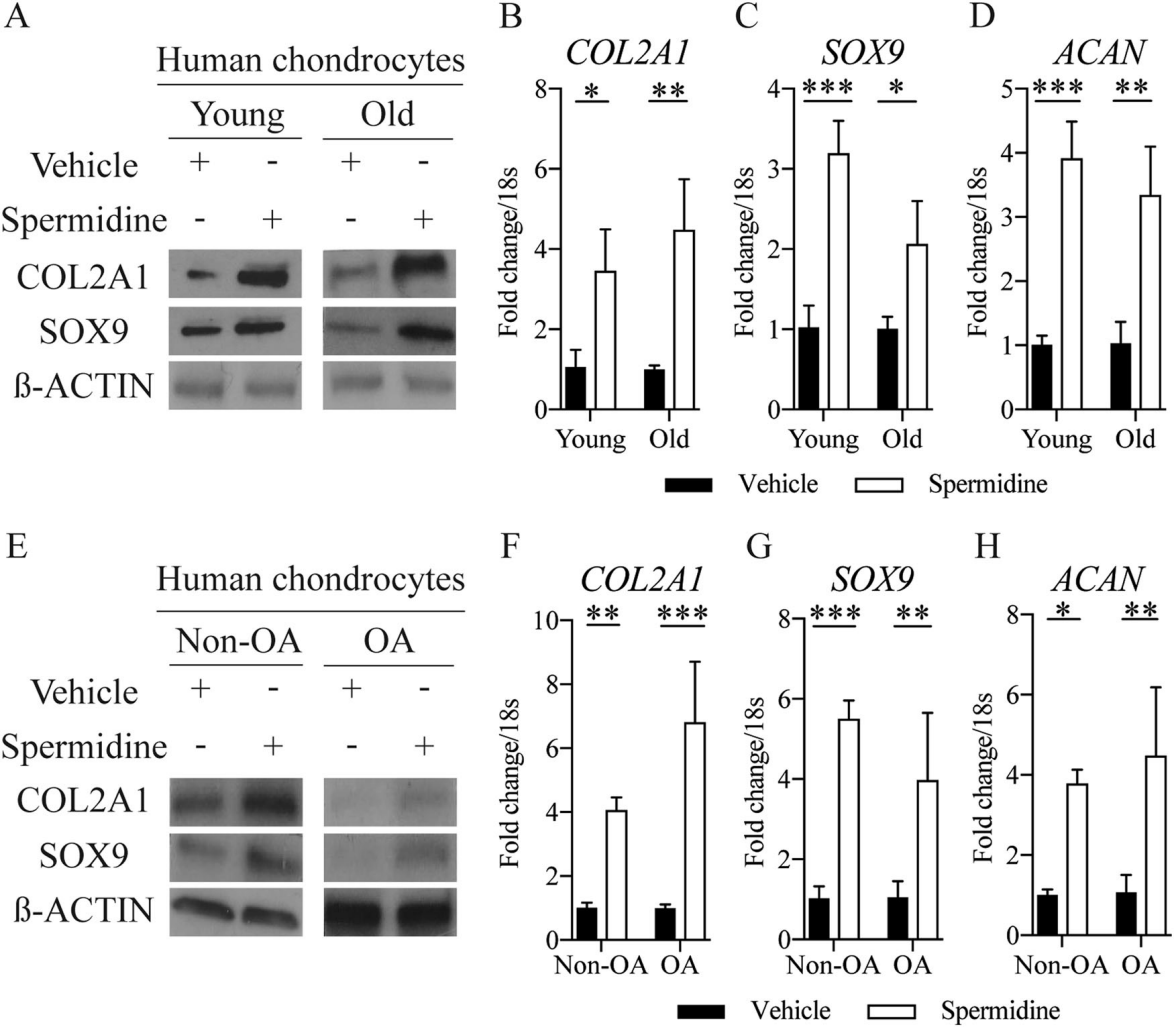
Spermidine increases the expression of key chondrogenesis markers. **a** Protein expression and (**b–d**) gene expression (RT-qPCR) of key chondrogenesis markers in isolated chondrocytes from young (21**–**37 years) and old (62**–**68 years) human knee joints treated with DMSO control or spermidine (100 nM) (*n* = 3). **e** Protein expression and (**f–h**) gene expression (RT-qPCR) of key chondrogenesis markers in isolated chondrocytes from non-OA control or OA human knee joints. Cells were either treated with DMSO control or spermidine (100 nM) for 2 h (*n* = 3). All RT-qPCR gene expressions were normalised to the endogenous level of 18s. All data are expressed as mean ± S.E.M of *n* observations. Students unpaired *t*-test or ANOVA with Tukeys comparison were used for statistical analysis. *p* < 0.05, *p* < 0.01 or *p* < 0.001 represented in all tables and Figures as *, ** or ***, respectively. NS  non–significant

We also sought to understand if important chondrogenesis markers (COL2A1, AGGRECAN, SOX9) could be increased by spermidine alongside the expression of autophagy markers. To our surprise, in young and old human and murine chondrocytes and in human isolated OA chondrocytes, we observed significantly elevated markers of chondrogenesis in spermidine-treated groups compared to vehicle-treated controls (Fig. 3a–h and Supplementary Figure 2j-m). These indicate that spermidine may induce a potential repair response in cartilage by activating autophagy and key chondrogenesis markers.

### Spermidine activates chondrocyte autophagy via EP300

**Fig. 4:**
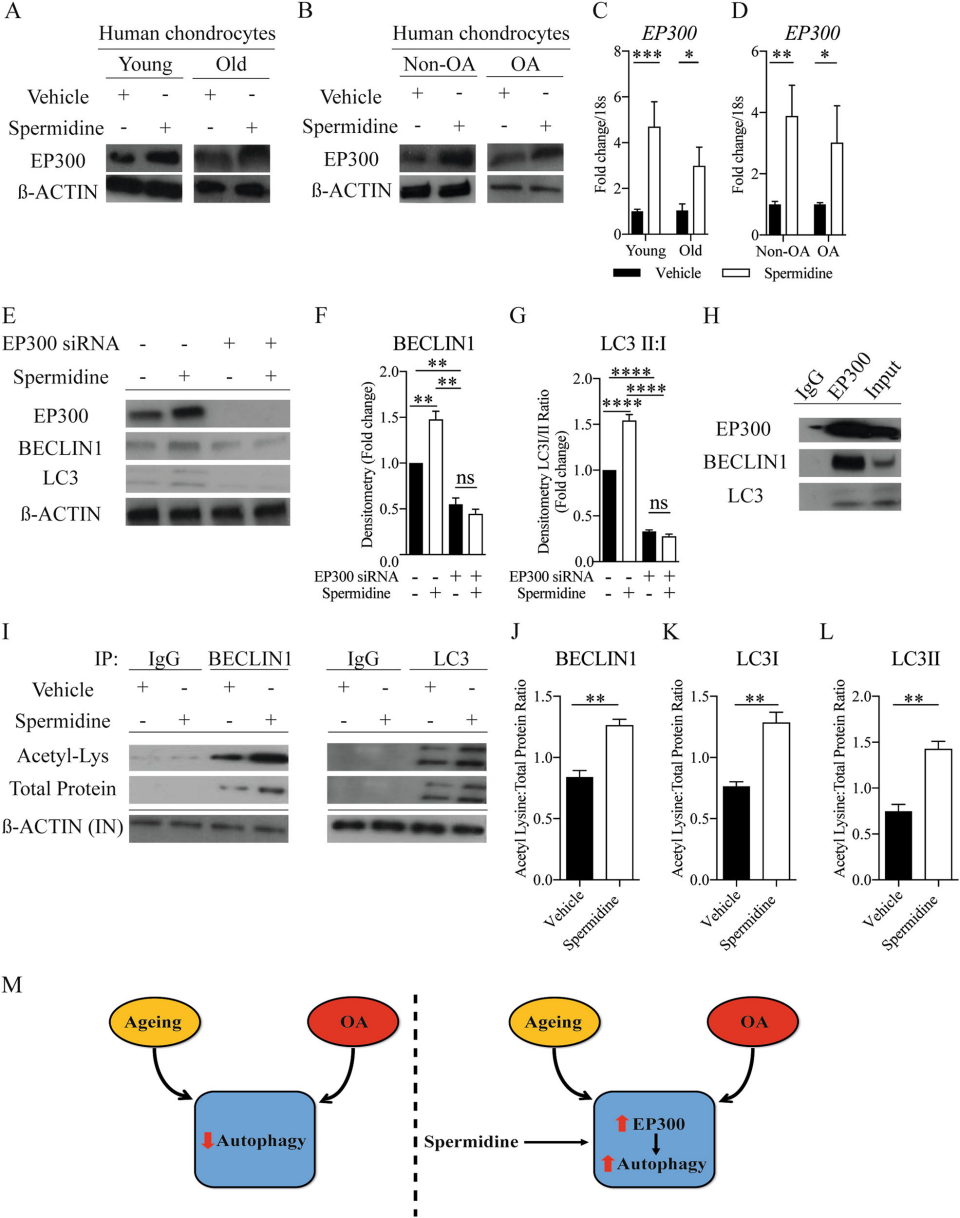
Spermidine activates chondrocyte autophagy via EP300. EP300 protein expression in (**a**) isolated chondrocytes from young (21–37 years) and old (62–68 years) human knee joints (*n* = 3) or (**b**) from non-OA control and OA human knee joints (*n* = 3) treated with DMSO control or spermidine (100 nM) for 2 h. EP300 gene expression (RT-qPCR) (**c**) in isolated chondrocytes from young (21–37 years) and old (62–68 years) human knee joints (*n* = 3) or (**d**) in non-OA control and OA human knee joints (*n* = 3) treated with DMSO control or spermidine (100 nM) for 2 h. **e** Representative western blot (**f and g**) densitometry quantification of autophagy protein expressions in chondrocytes following EP300 siRNA or control siRNA transfection +/− spermidine (100 nM) or DMSO treatment for 2 h (*n* = 3). **h** Representative western blot showing the binding of autophagy markers using immunoprecipitation assays against EP300 in HTB-94 cells. **i** Western blots showing representative acetylation status (total acetyl lysine) and total protein expression of immunoprecipitated (IP) BECLIN1 and LC3 in HTB-94 cells treated with DMSO (control) or spermidine (100 nM) for 2 h. β-Actin from input (IN) lysate is shown as loading control. **j**–**l** Ratio of acetyl lysine compared to total protein of autophagy markers in HTB-94 cells treated with DMSO (control) or spermidine (100 nM) for 2 h (*n* = 3). **m** Ageing and OA leads to impaired regulation of autophagy. Pharmacologically targeting this impairment with spermidine upregulates chondrocyte autophagy via EP300. All RT-qPCR gene expressions were normalised to the endogenous level of 18s. All data are expressed as mean ± S.E.M of *n* observations. Students unpaired *t*-test or ANOVA with Tukeys comparison were used for statistical analysis. *p* < 0.05, *p* < 0.01, *p* < 0.001 or *p* < 0.0001 represented in all tables and Figures as *, **, ***, or ****, respectively. *NS*  non–significant

Previous studies have demonstrated that spermidine can activate autophagy via the acetyltransferase EP300^8,13^. Hence, we sought to determine whether spermidine activates (and re-activates) autophagy in chondrocytes by interacting with EP300 in this system. Following spermidine treatment, gene and protein expression of EP300 increased in human and murine young and old isolated chondrocytes and in human OA chondrocytes, compared to respective untreated control cells (Fig. 4a–d and Supplementary Figure 3a and b). To validate if spermidine activates chondrocyte autophagy via EP300, we  silenced EP300 in chondrocytes prior to spermidine treatment. This led to the drug failing to increase the expression of autophagy proteins and, interestingly, knockdown of EP300 decreased expression of autophagy proteins beclin 1 and LC3 compared to control cells (Fig. 4e–g). Immunoprecipitation analysis determined that EP300 directly interacts with autophagy-related proteins, beclin 1 and LC3 (Fig. 4h). Moreover, both BECLIN1 and LC3 proteins were functionally modified by spermidine treatment, showing increased acetylation of lysine residues associated with enhanced protein activity and total protein expression overall (Fig. 4i–m).

## Discussion

Collectively, our data suggest the decreasing levels of autophagy and polyamine synthesis occur in ageing and OA chondrocytes, and can be replenished by spermidine. Polyamines are polycations that are found in cells in many organisms and have been shown to decrease during ageing^7^. Transgenic animals overexpressing ODC and SSAT, the enzymes responsible for polyamine synthesis, demonstrated increased ageing phenotypes^14^. These findings support our data where a decrease in polyamine synthesis was observed in aged chondrocytes compared to young cells and in OA samples compared to non-OA samples. The decrease in enzymes-related to polyamine synthesis was accompanied directly by a decrease in autophagy. Previous studies have shown how decreased chondrocyte autophagy leads to cartilage damage and exacerbated OA^4,6,15,16^. Hence, we explored whether replenishing the deficiency of polyamines in aged chondrocytes can activate autophagy.

Spermidine is a naturally occurring compound, found in high concentrations in peas, brown rice and beans and decreases in expression during ageing^7^, whilst supplementation with spermidine increases lifespan in lower organisms^9^. Our data indicate spermidine increases autophagy in both young and old chondrocytes, indicating that the lower levels of polyamine-related enzymes in aged chondrocytes might be reversible when such cells are supplied with the correct stimuli, to restore adequate cellular function. However, OA-derived chondrocytes are typically aberrant and unresponsive. Surprisingly, spermidine increased autophagy in cells from both non-OA and OA patients, and indicating the potential to use polyamines to activate autophagy, and thereby reduce the development of OA. Previously, Carames et al.^15^ demonstrated the autophagy activator rapamycin protected against cartilage damage in vivo. However, unlike spermidine, rapamycin has toxicity concerns and many off-target mechanisms, including immunosuppression in humans^17–19^. Spermidine also increased markers of chondrogenesis in both healthy (young and old) and OA chondrocytes. These data may suggest polyamines could be used to promote repair or regenerate cartilage tissue.

Spermidine has previously been shown to function (in part) via activation of the EP300 acetyltransferase^8,9^. We noticed an increase in EP300 expression after spermidine treatment of chondrocyte, and simultaneous to autophagy activation, whereas silencing EP300 reduced autophagy-related protein expression, suggesting that EP300 is regulating spermidine-induced autophagy in this system. As a component of the TIP60 complex, EP300 has been shown to modify acetylation of autophagy-related proteins in lower organisms^20^, supporting our finding that EP300 binds directly to autophagy proteins to increase their acetylation status and activity in mammalian chondrocytes. Interestingly, spermidine has previously been used in vivo to enhance autophagy in a dose-dependent manner and protect against myopathy^21^, neurodegeneration^22^, and age-related defects in T cell response^23^. Further pre-clinical studies are required to investigate whether spermidine could prevent OA development in experimental animal models of arthritic disease.

Together our data indicate that polyamine synthesis is reduced in chondrocytes during ageing and replenishing this defect by spermidine treatment activates autophagy and promotes chondrogenesis. We have identified new direct interactions between the epigenetic modifier EP300 and autophagy-related proteins, where functional post-translational modifications occur to increase chondrocyte autophagy. Targeting autophagy pharmacologically with spermidine or other polyamines may therefore represent a viable, cost-effective and well-tolerated approach to the management of OA.

## Electronic supplementary material

Supplementary Materials
