# Supplementary material for: Spermidine restores dysregulated autophagy and polyamine synthesis in aged and osteoarthritic chondrocytes via EP300
Source: Exp Mol Med. 2018 Sep 19;50(9):123. doi: 10.1038/s12276-018-0149-3 (PMC6145946; doi:10.1038/s12276-018-0149-3)
Supplement: Supplementary file 1 — Supplementary Materials [file 12276_2018_149_MOESM1_ESM.pdf]

Supplementary figure 1

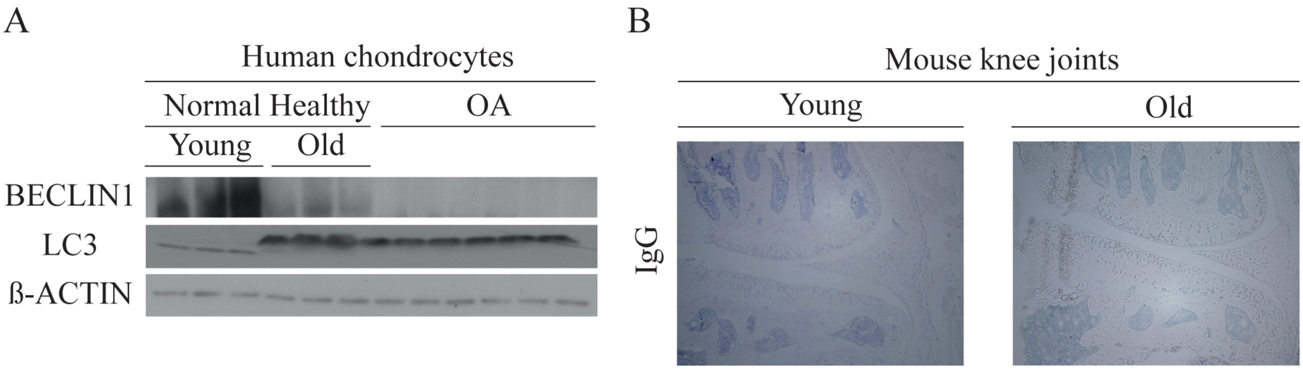

Supplementary Figure 1 - Autophagy markers *in vivo*.

(A) Autophagy protein expression in isolated chondrocytes from young healthy (21-37yrs), old (62-68yrs) and OA (49-86yrs) knee joints. **(B)** Representative images (x20) of rabbit IgG (used in immunohistochemistry experiment) control staining of knee joints from WT mice at young (2 months) or old (16 months) of age.

Supplementary figure 2

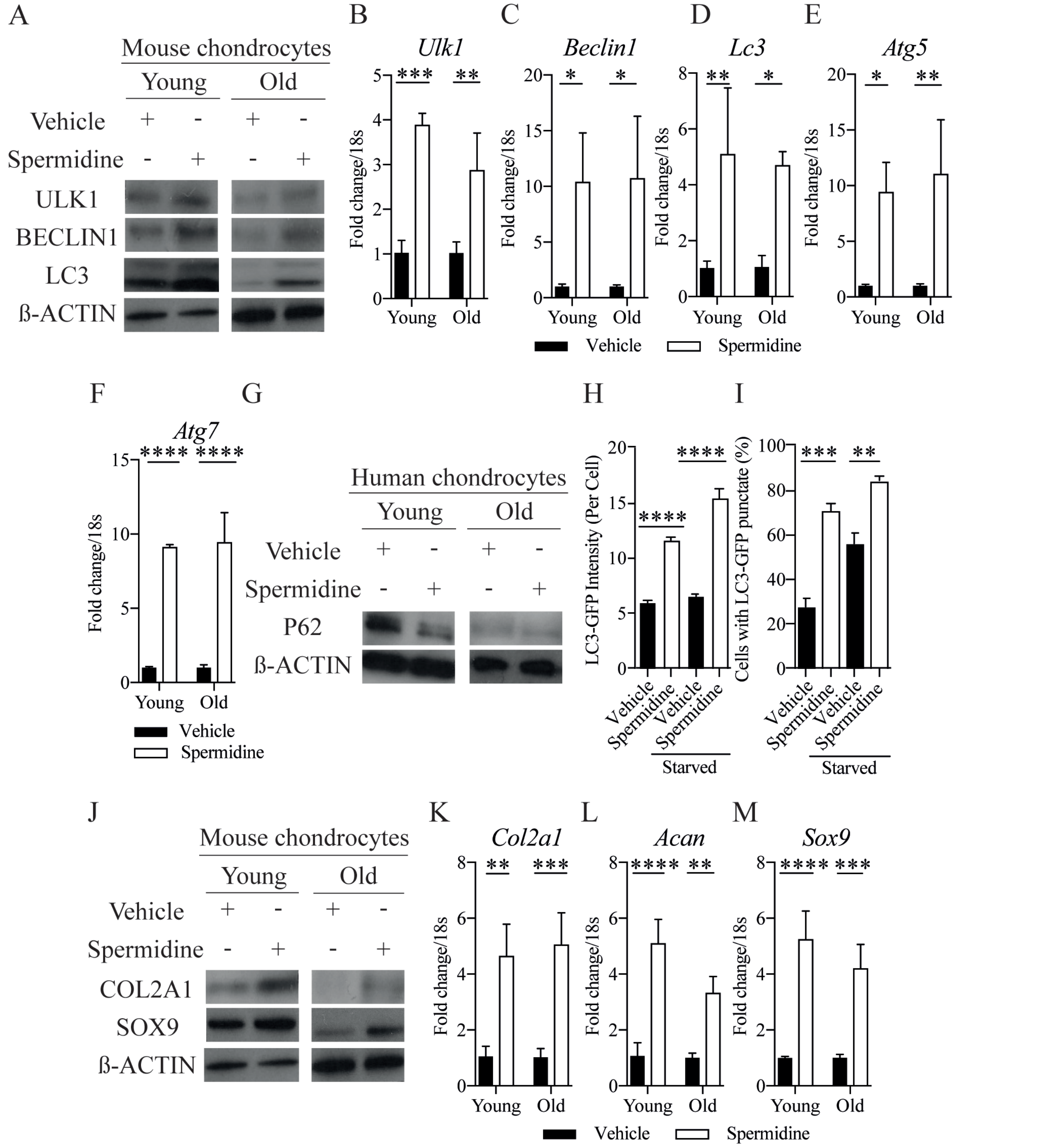

Supplementary Figure 2 - The effect of spermidine treatment on autophagy and chondrogenesis markers. (A) Representative western blot and **(B-F)** gene expression (RT-qPCR) of key autophagy proteins in avulsed femoral heads from WT young (2 months) or old (16 months) mice treated with either spermidine (100 nM) or DMSO treatment for 2 h (*n*=3). **(G)** Representative western blot showing P62 protein expression in isolated chondrocytes from young (21-37 yrs) and old (62-68 yrs) knee joints treated with DMSO control or spermidine (100 nM) for 2 h. **(H)** Qualification of total LC3-GFP intensity and **(I)** percentage of chondrocytes with LC3 positive punctate from avulsed femoral heads from LC3-GFP mice treated with either DMSO control or spermidine (100 nM) for 2 h before fixation in serum or serum free media (*n*=15-37 cells from 3 mice per treatment group). **(J)** Representative western blot and **(K-M)** gene expression (RT-qPCR) of key chondrogenesis markers in avulsed femoral heads from WT young (2 months) or old (16 months) mice treated with either spermidine (100 nM) or DMSO treatment for 2 h (*n*=3). All RT-qPCR gene expressions were normalised to the endogenous level of 18s. All data are expressed as mean ± S.E.M of *n* observations. ANOVA with Tukeys comparison was used for statistical analysis. NS= non–significant. p< 0.05, p< 0.01, p< 0.001 or p< 0.0001 represented in all tables and Figures as *, **, *** or **** respectively.

Supplementary figure 3

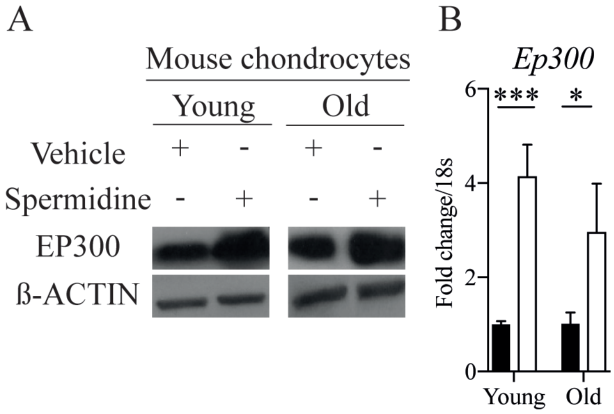

Supplementary Figure 3 - Spermidine activates EP300 in murine chondrocytes. (A) Representative western blot and **(B)** gene expression (RT-qPCR) of EP300 in avulsed femoral heads from WT young (2 months) or old (16 months) mice treated with either spermidine (100 nM) or DMSO treatment for 2 h (*n*=3). RT-qPCR gene expression was normalised to the endogenous level of 18s. All data are expressed as mean ± S.E.M of *n* observations. ANOVA with Tukeys comparison was used for statistical analysis. $p < 0.05$ or $p < 0.001$ represented in Figures as * or *** respectively.
